# Unique Substrates Secreted by the Type VI Secretion System of *Francisella tularensis* during Intramacrophage Infection

**DOI:** 10.1371/journal.pone.0050473

**Published:** 2012-11-20

**Authors:** Jeanette E. Bröms, Lena Meyer, Kun Sun, Moa Lavander, Anders Sjöstedt

**Affiliations:** Department of Clinical Microbiology, Clinical Bacteriology and Laboratory for Molecular Infection Medicine Sweden, Umeå University, Umeå, Sweden; Cornell University, United States of America

## Abstract

Gram-negative bacteria have evolved sophisticated secretion machineries specialized for the secretion of macromolecules important for their life cycles. The Type VI secretion system (T6SS) is the most widely spread bacterial secretion machinery and is encoded by large, variable gene clusters, often found to be essential for virulence. The latter is true for the atypical T6SS encoded by the *Francisella* pathogenicity island (FPI) of the highly pathogenic, intracellular bacterium *Francisella tularensis*. We here undertook a comprehensive analysis of the intramacrophage secretion of the 17 FPI proteins of the live vaccine strain, LVS, of *F. tularensis*. All were expressed as fusions to the TEM β-lactamase and cleavage of the fluorescent substrate CCF2-AM, a direct consequence of the delivery of the proteins into the macrophage cytosol, was followed over time. The FPI proteins IglE, IglC, VgrG, IglI, PdpE, PdpA, IglJ and IglF were all secreted, which was dependent on the core components DotU, VgrG, and IglC, as well as IglG. In contrast, the method was not directly applicable on *F. novicida* U112, since it showed very intense native β-lactamase secretion due to FTN_1072. Its role was proven by ectopic expression *in trans* in LVS. We did not observe secretion of any of the LVS substrates VgrG, IglJ, IglF or IglI, when tested in a FTN_1072 deficient strain of *F. novicida*, whereas IglE, IglC, PdpA and even more so PdpE were all secreted. This suggests that there may be fundamental differences in the T6S mechanism among the *Francisella* subspecies. The findings further corroborate the unusual nature of the T6SS of *F. tularensis* since almost all of the identified substrates are unique to the species.

## Introduction

Gram-negative bacteria have evolved various types of sophisticated machineries specialized for the secretion of macromolecules as a means to promote bacterial fitness and/or establish colonization or attachment to host cells. Out of the seven secretion machineries identified so far, the Type VI secretion system (T6SS) is the most recently identified. It occurs widely in both pathogenic bacteria and in commensals and is encoded by large, variable gene clusters which are characterized by a core of ∼ 13 highly conserved components believed to form the basic trans-envelope secretion apparatus, and, in addition, also some accessory components [1], [2], [3]. T6SSs have been implicated in the virulence of pathogens such as *Burkholderia mallei*, *Vibrio cholera, Salmonella typhimurium*, and *Edwardsiella tarda*, and are central for functions like quorum sensing and stress responses, biofilm formation, symbiosis, resistance to amoeba predation and phagocytosis, mouse virulence, intramacrophage growth, and anti-bacterial activities [4], [5], [6], [7], [8], [9], [10], [11]. Possibly, the latter is important for the establishment of many types of infection, since it allows pathogens to successfully compete in polymicrobial sites, such as the gastro-intestinal tract.

Common to many T6SSs is the secretion of Hcp and VgrG into the extracellular medium or into target cells. These highly conserved substrates share substantial structural resemblance to the tail tube and needle complex of T4 bacteriophages, respectively, which are required to puncture host membrane in the context of phage infection [12], [13], [14]. Intriguingly, several of the core components of T6SSs also share similarities with bacteriophage structures, such as the base-plate or sheath components, suggesting that these machineries are evolutionary related (reviewed in [15]). In *V. cholerae*, VipA/VipB have been shown to form tubular structures that structurally resemble bacteriophage T4 contractile tail sheath [14], [16] and a recent, elegant microscopy study revealed that the similarity also extends to the level of function, as the tubules were shown to cycle between assembly, quick contraction, disassembly, and re-assembly [17]. This suggests that the sheath may energize the translocation of substrates into the extracellular milieu or into adjacent target cells by a phage tail-like contraction mechanism.

Hcp and VgrG are not only structural components of the T6SS, but may also possess effector functions. In the case of VgrGs, these functions can be attributed to C-terminal extensions that upon delivery into the host cell interfere with cellular functions. For example, the Rtx domain of *V. cholera* VgrG1 cross-links actin [12], while the VIP-2 domain of *A. hydrophila* VgrG1 possesses actin-APD-ribosylation activity [18]. In addition, some VgrGs carry domains that exhibit homology to bacterial cell-wall degrading enzymes, proteases as well as bacteriocins, suggesting that these proteins may have a bactericidal function [19], [20], [21]. In *A. hydrophila*, Hcp was shown to bind to the surface of macrophages and to induce IL-10 and TGF-β production, which resulted in impaired recruitment and inhibition of phagocytosis [22]. In addition, Hcp proteins with C-terminal extensions have been identified in *S. enterica* and *E. coli*, which may represent evolved Hcp proteins with effector functions [20], [23], [24]. Besides VgrG and Hcp, only a few T6SS-secreted proteins have been identified, most notably EvpP of *E. tarda* [25], the bactericidal Tse1-3 system, secreted by the HSI-1 T6SS of *P. aeruginosa* [26], and the Tae2 (type VI amidase effector) of *Burkholderia thailandensis*, which is important for growth competition against other bacteria [27]. In the latter study, 11 potential substrates secreted by the T6SS-1 system of *B. thailandensis* were identified, many of which may be required for mediating interbacterial interactions [27].

An aberrant variant of T6SSs is found in the highly virulent, facultative intracellular bacterium *Francisella tularensis*. Little is known about the molecular mechanisms of *Francisella* pathogenesis, but its ability to survive and replicate within macrophages appears intimately linked to its virulence [28]. Within this normally hostile cell type, the *Francisella*-containing phagosome appears to evade lysosome fusion and relatively quickly, the bacterium escapes into the cytoplasm and thereafter starts to proliferate [29], [30], [31]. The intramacrophage replication is dependent on a multitude of proteins, many of which are encoded by the *Francisella* pathogenicity island (FPI). This is a large, duplicated 33-kb region and a phylogenetic analysis has revealed that it constitutes the lone member of a distantly related fifth group of T6SSs [1]. Essentially all of the FPI proteins are conserved among the *F. tularensis* subspecies, and most of them are essential for intracellular replication as well as growth within the amoeba *Acanthamoeba castellanii*, a putative reservoir of *F. tularensis* (reviewed in [32]). The FPI encodes a truncated form of VgrG that forms multimers consistent with its suggested role as a trimeric needle complex [33]. During intracellular infection, VgrG as well as IglI, a substrate unique to *Francisella*, is secreted into the macrophage cytosol [34], [35]. While secretion of VgrG occurred independently of the FPI, export of IglI was dependent on the FPI for *F. tularensis* subsp. *novicida* strain U112, but not for *F. tularensis* LVS, the live vaccine strain [34], [35].

To further identify potential substrates among the FPI proteins, we undertook a comprehensive analysis of the intramacrophage secretion of FPI proteins by the LVS strain. All of the 17 FPI proteins were expressed as fusions to TEM beta-lactamase, which together with the fluorescent substrate CCF2-AM, allowed us to follow their secretion into the macrophage cytosol over time. By this means, significant secretion of IglE, IglC, VgrG, IglI, PdpE, PdpA, IglJ and IglF was observed resulting in the production of blue fluorescence of infected cells. In all cases, secretion was dependent on the core components DotU, VgrG, and IglC as well as IglG. The findings further emphasize the unusual nature of the T6SS of *F. tularensis* and its distant relationship to other T6SS, since all identified substrates, except for VgrG, are unique to the species.

## Results

### Construction of FPI protein TEM β-lactamase fusions

In order to identify putative FPI protein substrates that are translocated by *F. tularensis* LVS during infection, we employed a fluorescent-based β-lactamase (TEM) translocation assay, which has previously been used to identify substrates of both Type III- and Type IV-secretion systems [36], [37], [38], [39], [40]. In this assay, each candidate gene is fused to TEM (β-lactamase) of *E. coli* and the bacterial strain expressing the fusion protein is used to infect host cells, which are then loaded with CCF2 substrate. Delivery of the β-lactamase fusion protein into host cell cytosol leads to cleavage of the substrate, resulting in an easily detectable change in fluorescence from green to blue emission.

**Figure 1:**
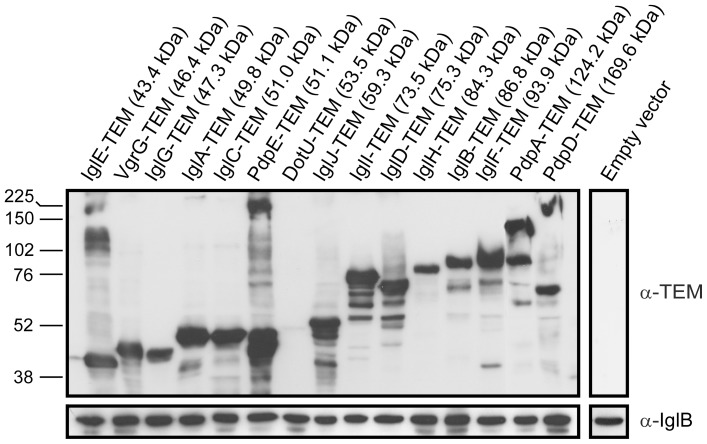
Production of FPI-TEM fusion proteins in LVS. Total cell lysates of *Francisella* LVS harboring various FPI-TEM fusions or an empty vector control were prepared and examined by Western-blot analysis using an antibody against TEM β-lactamase (top panel) or IglB (bottom panel). The latter was used as a loading control. The Full-range rainbow molecular weight marker from Amersham was used in the analysis, and the sizes of the marker are indicted. The molecular weight indicated for each of the fusion protein was deduced from the primary sequence by using the SAPS server (www.ebi.ac.uk/Tools/saps/).

To generate FPI protein-TEM fusions, we first constructed vector pJEB709, which encodes the mature β-lactamase from *E. coli* under the control of the constitutive *groE* promoter. Individual FPI genes were amplified by PCR and inserted into pJEB709 to generate translational C-terminal fusions with the downstream β-lactamase gene. We expressed the constructs in LVS instead of their isogenic mutant background to overcome the problems with lack of complementation exhibited by many of the chimeras. In fact, out of 10 TEM-constructs that we specifically tested (IglE, VgrG, IglF, IglH, DotU, IglJ, IglD, IglC, IglB, IglA), only 3 (IglA, IglD, IglJ) were able to complement the corresponding mutant for growth in macrophages and/or LDH release (data not shown). Since expression of the wild-type proteins without a tag generally leads to phenotypic complementation of FPI mutants [34], [35], [41], [42], this suggests that the 29.5 kDa TEM-tag sterically interferes with protein function. To verify that the chimeras were indeed expressed, we used TEM β-lactamase antibodies. Although the level of expression varied to some extent, a protein corresponding to the expected size of the fusion was detected in most of the samples with the exceptions of the TEM fusions of IglG and IglD, which both were somewhat smaller than their predicted size (Fig. 1). Moreover, DotU-TEM was barely detected in the bacterial pellets, while the two largest fusion proteins, PdpB-TEM and PdpC-TEM, could not be detected at all, suggesting that they may be unstable (Fig. 1 and data not shown). Since LVS encodes a truncated form of PdpD, we expressed the full-length protein from *F. novicida* strain U112 instead (Fig. 1).

### Identification of proteins transferred by the Type VI secretion system of *Francisella tularensis* LVS

**Figure 2:**
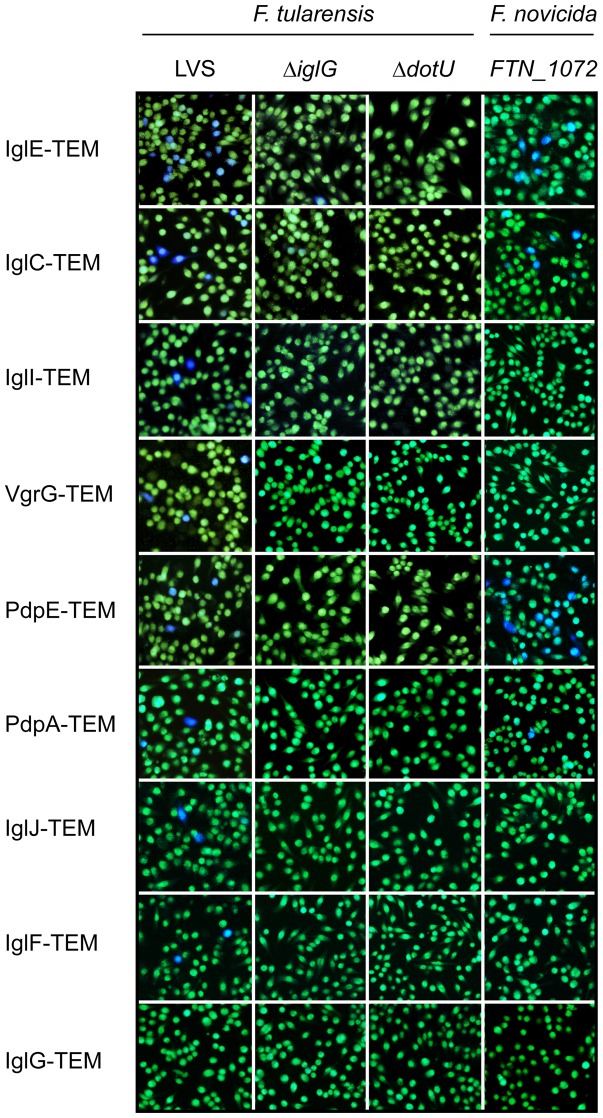
Secretion of *Francisella* FPI proteins into J774A.1 macrophages. Macrophages were infected either with LVS, mutants thereof or an *F. novicida FTN_1072* mutant expressing different FPI-TEM fusions. After infection, cells were washed and loaded with CCF2/AM and analyzed using live cell microscopy. TEM β-lactamase activity is revealed by the blue fluorescence emitted by the cleaved CCF2 product, whereas uncleaved CCF2 emits a green fluorescence.

After verifying the expression of the fusion proteins in *F. tularensis*, we infected J774 macrophages with *F. tularensis* strains expressing the β-lactamase fusions. At indicated time points, cells were loaded with the CCF2 substrate in the presence of the drug Probenicid to prevent the substrate from being excreted by the cells. As positive and negative controls, we used LVS expressing VgrG-TEM or IglG-TEM, respectively, as we have previously shown that a CyaA fusion of VgrG, but not IglG, is secreted into macrophages during infection [34]. Translocation of the β-lactamase chimeras as reflected by the presence of cells emitting blue fluorescence signals was assessed using a live-cell microscope. At 18 h post-infection, infection with LVS alone resulted in cells emitting green fluorescence only, suggesting that the endogenous β-lactamases encoded by LVS, FTL_0879 and FTL_0957, were not secreted/and or able to cleave the β-lactam ring of CCF2-AM (data not shown), a prerequisite for the use of the assay. Similarly, no blue fluorescence was detected when cells were infected with LVS expressing IglG-TEM (Fig. 2), suggesting that it was an appropriate negative control. In contrast, infection with LVS expressing VgrG-TEM resulted in a significant, albeit small population of blue fluorescent cells (2.83±0.15%), suggesting that VgrG was secreted during infection (Fig. 2). This supports our and others' previous results using a CyaA reporter-based approach [34], [35]. Out of the 16 ORFs predicted to encode full-length proteins in LVS, as well as *F. novicida*-derived PdpD, a total of 8 proteins that consistently promoted secretion of TEM were identified and the numbers of blue fluorescent cells and SEM obtained with these constructs at 18 h were: 11.9±0.72% (IglE-TEM), 10.6±0.52% (IglC-TEM), 2.83±0.15% (VgrG-TEM), 2.52±0.20% (IglI-TEM), 2.36±0.17% (PdpE-TEM), 1.81±0.19% (PdpA-TEM), 1.28±0.12% (IglJ-TEM) and 1.12±0.08% (IglF-TEM) (Fig. 2 and Table 1). By comparing secretion of these substrates at different time points, 3 h, 9 h, 18 h, and 24 h, we concluded that secretion was most prominent at the interval of 18–24 h, therefore, we focused our studies on the 18 h time point (Table 1 and data not shown). In general, there was no clear correlation between the overall levels of the fusions and translocation efficiencies (Figs. 1 and 2, Table 1). Therefore, the level of fusion protein expressed in *F. tularensis* appears to play a minor role for the translocation efficiency of a particular substrate. The 8 proteins do not share any detectable common features, except that they are fairly small proteins (five have M_w_ of 14.5–30.4 kDa according to SAPS (www.ebi.ac.uk/Tools/saps/)), IglI (44.6 kDa), IglF (65.0 kDa) and PdpA (95.3 kDa) being the exceptions. Among the substrates, IglI and VgrG had previously been reported to constitute FPI-encoded substrates during intramacrophage infection, however, surprisingly, their secretion was reported to occur independently of the FPI in LVS and, in the case of VgrG, also in the *F. tularensis* subsp *novicida* strain U112 [34], [35]. To determine whether secretion of the TEM hybrids also occurred in an FPI-independent fashion, constructs encoding fusions exhibiting detectable translocation were introduced into the mutant strains Δ*dotU*, Δ*vgrG*, Δ*iglC* and *iglG*. The first three encode core components of the *F. tularensis* T6SS [30], [33] and are therefore likely to be essential for translocation. In contrast to these, Δ*iglG* exhibits wild-type levels of replication in J774 macrophages and therefore the number of bacteria will not be a limiting factor for detection of protein translocation. The resulting strains were tested for delivery of the β-lactamase FPI fusions into host cells. None of the fusions caused detectable translocation in the Δ*dotU*, Δ*vgrG* or Δ*iglC* backgrounds, and none or very dramatically reduced translocation in the Δ*iglG* background (Fig. 2 and Table 1). Thus, secretion is dependent on the FPI-encoded components DotU, VgrG, IglC and IglG. Therefore, these are likely to encode structural components of the translocation machinery and strongly suggest that secretion of FPI proteins is indeed FPI-dependent.

**Table 1:** Secretion of FPI-TEM fusions upon J774A.1 infections.

Percentage of blue fluorescent cells ± SEM in different bacterial backgrounds												
Time point	3 h	9 h		18 h	18 h		18 h		18 h		18 h	18h
Strain	*F. tularensis* a											*F. novicida* a
	LVS		LVS	LVS		Δ*iglG*		Δ*dotU*		Δ*vgrG*	Δ*iglC*	*FTN_1072*
IglE-TEM	1.84±0.23***		6.03±1.21***	11.9±0.72***		BC		BC		BC	BC	3.48±0.32***
IglC-TEM	BC		0.50±0.11	10.6±0.52***		0.66±0.16*		BC		BC	BC	2.64±0.16***
VgrG-TEM	BC		BC	2.83±0.15***		BC		BC		BC	BC	BC
IglI-TEM	BC		BC	2.52±0.20***		BC		BC		BC	BC	BC
PdpE-TEM	BC		1.30±0.17***	2.36±0.17***		BC		BC		BC	BC	16.9±1.33***
PdpA-TEM	BC		BC	1.81±0.19***		BC		BC		BC	BC	0.70±0.09***
IglJ-TEM	BC		BC	1.28±0.12***		BC		BC		BC	BC	BC
IglF-TEM	BC		BC	1.12±0.08***		BC		BC		BC	BC	BC
IglG-TEM	NT		NT	BC		BC		BC		BC	BC	BC

a The percentage of blue fluorescent cells was significantly different for infections with strains expressing a FPI-TEM fusion *in trans* compared to infections with the parental strains only (*, *P*<0.05; ***, *P*<0.001). BC  =  below the cut-off of the assay, *i.e.*<0.5%. NT  =  not tested.

### Beta-lactamase secretion in F. tularensis subsp. novicida U112

**Figure 3:**
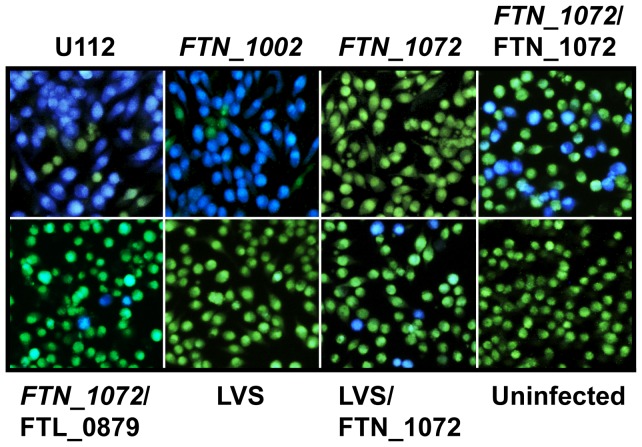
Secretion of *Francisella* beta-lactamases into J774A.1 macrophages. Macrophages were infected with strains of *F. tularensis* or *F. novicida* expressing beta-lactamase genes *in cis* or *trans*. After infection, cells were washed and loaded with CCF2/AM and analyzed using live cell microscopy. β-lactamase activity is revealed by the blue fluorescence emitted by the cleaved CCF2 product, whereas uncleaved CCF2 emits a green fluorescence.

We also wanted to verify the TEM results using another species of *Francisella*. For this reason, we included the *F. novicida* strain U112 in our study. To our surprise, infection with U112 alone using the identical experimental setup resulted in 56.1±1.8% of blue fluorescent cells, suggesting that *F. novicida* U112, in contrast to LVS, encodes native β-lactamase(s) that is/are secreted and capable of cleaving CCF2 (Fig. 3). According to the *Francisella* genome database, U112 harbors two β-lactamase genes, *i.e. FTN_1002* and *FTN_1072*, which are homologous to *FTL_0957* and *FTL_0879* of LVS, respectively. To determine which, if any, of these genes was responsible for the efficient cleavage of the CCF2 substrate, we included clones from a two-allele transposon mutant library [43] with insertions in either *FTN_1002* or *FTN_1072*. The gene responsible for the blue fluorescence during infection was found to be *FTN_1072*, as the corresponding insertion mutant dramatically reduced the amount of blue cells to values below the cut-off of the assay (<0.5%), while insertion mutations within gene *FTN_1002* had no obvious effect (Fig. 3).

**Figure 4:**
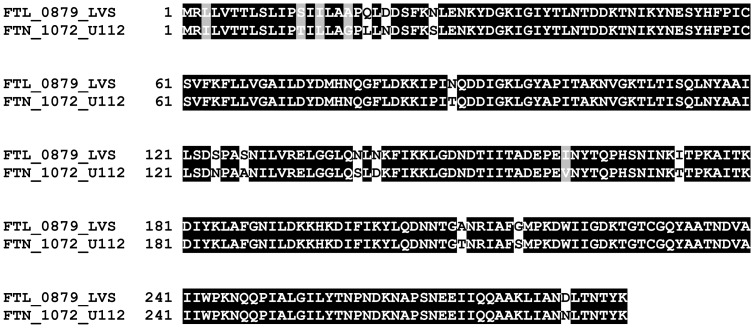
Alignment of β-lactamases FTL_0879 and FTN_1072 from LVS and U112 respectively. Alignments were generated using the ClustalW2 web server (http://www.ebi.ac.uk/Tools/clustalw2/index.html) and areas of amino acid identity (black boxes) or similarity (grey boxes) illustrated using the BOXSHADE 3.21 web server (http://www.ch.embnet.org/software/BOX_form.html).

From an alignment of FTN_1072 and FTL_0879, it became apparent that there are many substitutions within the LVS homologue, which may account for the inability of FTL_0879 to cleave CCF2 (Fig. 4). While altered specificity may be one explanation to these differences, another possibility is that secretion in general may be much more efficient in U112 compared to LVS. While Bina *et al* have shown that the encoded product of *FTL_0879* is indeed secreted in LVS [44], there are no studies where the secretion efficiencies of FTN_1072 and FTL_0879 have been directly compared. To differentiate between these two possibilities, we therefore expressed *FTN_1072* or *FTL_0879 in trans* in the *F. novicida FTN_1072* mutant. While expression of the former partially restored secretion upon infection with the *FTN_1072* mutant, resulting in 25.9±0.91% of blue fluorescent cells (∼46% of U112-levels; *P<*0.001) (Fig. 3), expression of LVS-derived *FTL_0879* resulted in only 2.79±0.77% of blue fluorescent cells (∼5% of U112-levels; *P<*0.001) (Fig. 3). These data clearly indicate that FTL_0879 is less potent at cleaving CCF2. If altered substrate specificity is the only explanation for the observed differences in CCF2 cleavage by *F. novicida* and LVS, we would expect that infection with LVS expressing *FTN_1072 in trans* would result in the same amount of blue fluorescent cells as for the complemented *F. novicida FTN_1072* mutant, however it turned out to be only 7.09±0.31%, *i.e.* 27% of *FTN_1072*/FTN_1072-levels (*P<*0.001) (Fig. 3). Thus, it appears as if also secretion of β-lactamases may be more efficient in *F. novicida* compared to LVS. To further investigate secretion in *F. novicida*, we introduced a selection of FPI-TEM fusions into the *FTN_1072* mutant and determined their translocation efficiencies during intramacrophage infections. The fusions included were IglE-TEM, IglC-TEM, VgrG-TEM, IglI-TEM, PdpE-TEM, PdpA-TEM, IglJ-TEM and IglF-TEM, which represented substrates secreted during an LVS infection, as well as IglG-TEM, which was not secreted by LVS. Using the TEM assay, we demonstrated secretion of PdpE, IglE, IglC, and PdpA, but not IglG, at 18 h and the numbers of blue fluorescent cells obtained with these constructs at 18 h were: 16.9±1.33% (PdpE), 3.48±0.32% (IglE), 2.64±0.16% (IglC) and 0.70±0.09% (PdpA) (Fig. 2 and Table 1). Surprisingly, however, in contrast to the findings on LVS, VgrG, IglI, IglJ and IglF were not secreted by *F. novicida* (Fig. 2. and Table 1). Thus, while secretion of *Francisella*-derived β-lactamases may occur more efficiently in *F. novicida-*infected cells than in LVS-infected cells, the same does not necessarily apply to FPI-TEM fusions. These results also suggest that PdpE, PdpA, IglE and IglC are common substrates of the T6SS of *Francisella* spp., but also that there are fundamental differences in the T6S mechanism of the different *Francisella* subspecies.

### The proton motive force impacts on T6S in F. tularensis

Recently, it has been unraveled that secretion of substrates by Type III secretion systems (T3SS) as well as flagella is dependent on the proton motive force (PMF) for export of proteins across the inner membrane [45], [46], [47], [48], while ATPases have been hypothesized to provide the initial energy required for substrate release and unfolding [47], [49]. In T6SSs, the ATPases IcmF and ClpV, the latter a member of the ClpB family of AAA+ ATPases, have been suggested to energize the secretion process [50]. The two ATPases are highly conserved in T6SSs, but the Walker A box commonly present in IcmF homologues is missing from the *F. tularensis* IcmF/PdpB. In addition, *F. tularensis* also appears to lack a ClpV homologue (reviewed in [32]). Therefore it is tempting to speculate that PMF may be the main energizer of the putative T6SS of *F. tularensis*.

**Figure 5:**
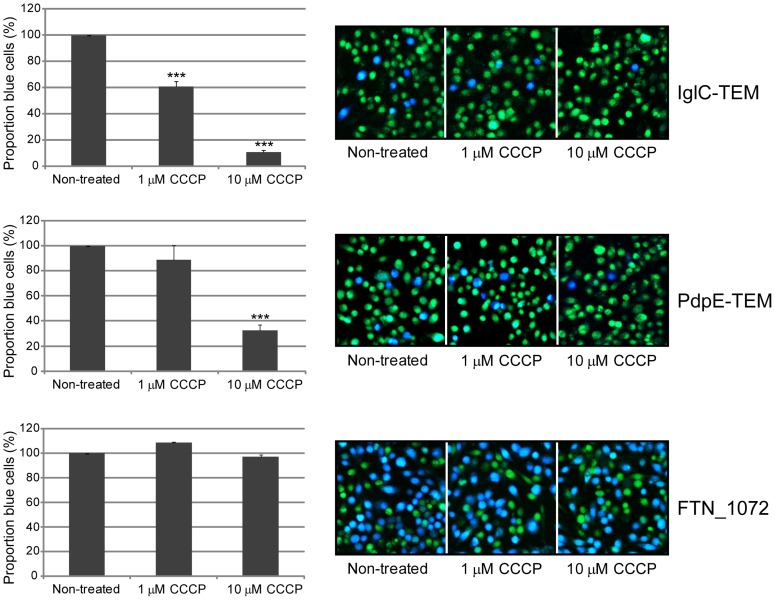
The impact of the PMF inbibitor CCCP on protein secretion in *F. tularensis*. Macrophages were infected with LVS expressing IglC-TEM *in trans*, *F. novicida FTN_1072* expressing PdpE-TEM *in trans* or U112 expressing native FTN_1072 beta-lactamase *in cis*. After infection, cells were washed and loaded with CCF2/AM and analyzed using live cell microscopy. β-lactamase activity is revealed by the blue fluorescence emitted by the cleaved CCF2 product, whereas uncleaved CCF2 emits a green fluorescence. The experiment was repeated 6 times using duplicate samples and a representative experiment is shown. The graphs demonstrate the average proportion of blue fluorescent cells of CCCP-treated samples *vs* non-treated samples and the standard error of the means (SEM). The asterisks indicate that the levels of blue fluorescent cells were significantly different for CCCP-treated samples compared to non-treated samples as determined by a 2-sided *t*-test with equal variance (***, *P*≤0.001).

To determine whether PMF plays a role in *F. tularensis* substrate export during infection, we used the membrane-permeable protonophore CCCP (carbonyl cyanide *m*-chlorophenylhydrazone), which is known to disrupt the PMF [51]. First, a potential toxic effect of CCCP was assessed by treating the LVS bacteria with different concentrations of the substance during growth in broth. Addition of 10 µM CCCP, a concentration previously shown to inhibit flagellar secretion in *Salmonella enterica* as well as Yop secretion in *Yersinia enterocolitica* [45], [46], [47], affected the growth of LVS in Chamberlain's medium, resulting in a 6 h delay before the culture reached lag-phase (Fig. S1). A small growth restriction was also seen in the presence of as little as 1 µM CCCP, although the delay before reaching lag-phase was only 1 h (Fig. S1). Importantly, growth resumed at essentially the initial rate upon removal of the compound after 3 h (data not shown). Therefore, under the conditions used, CCCP does not affect *F. tularensis* viability. To assess the effect of CCCP on T6S-mediated export in LVS, 1 or 10 µM of the substance was added at 0 h, and samples were analyzed for secretion of IglC-TEM after 18 h. CCCP was found to have a dose-dependent effect on the secretion of IglC-TEM. At a concentration of 1 µM, the numbers of blue fluorescent cells were reduced by ∼ 40% (*P<*0.001), while at the higher concentration they were ∼ 10% of the numbers of the non-treated control (*P<*0.001) (Fig. 5). Also a decrease in secretion of PdpE-TEM, when expressed *in trans* from the *F. novicida FTN_1072* mutant, was observed in the presence of CCCP, as 10 µM of the substance reduced the blue fluorescent population by 68% (*P*<0.001 *vs* the non-treated control) (Fig. 5). In contrast, CCCP had no significant impact on the secretion of the native FTN_1072 β-lactamase in the *F. novicida* strain U112 regardless of concentration (Fig. 5), suggesting that it specifically targets the export of the FPI substrates. Under the conditions tested, we were unable to detect any CCCP-mediated effect on intramacrophage growth or LDH release of LVS (Fig. S2), suggesting that the PMF does not contribute to these phenotypes, but plays an important role for T6S.

## Discussion

This study is the first comprehensive study of intracellular FPI protein secretion by *F. tularensis* and also the first *Francisella* study utilizing the TEM β-lactamase assay. There are a number of recent examples when this assay has been used for detecting intracellular translocation of bacterial proteins, even for high throughput screening of secretion, *e.g*., in *Legionella* and *Coxiella* [38], [39]. The T6SS of *F. tularensis* is poorly understood and the limited data on secretion obtained hitherto not fully compatible. Previous data was based on the use of the CyaA reporter, but were only focused on the roles of the PdpE, IglI and VgrG proteins [34], [35]. It was concluded that IglI and VgrG were both secreted in *F. novicida* and LVS, however, the data was conflicting regarding the requirement for other FPI proteins since the findings in *F. novicida* suggested that only secretion of IglI was FPI-dependent, whereas the findings on LVS concluded that IglI as well as VgrG secretion was FPI-independent [34], [35]. Our present investigation using the TEM fluorescent reporter assay demonstrated that none of the eight identified substrates, IglE, IglC, VgrG, IglI, PdpE, PdpA, IglJ and IglF, were exported in the absence of IglC, IglG or the T6SS core components DotU or VgrG. This indicates that the FPI indeed constitutes a secretion system and that the DotU and VgrG proteins, as their homologous core components of other T6SS, have the same essential functions for secretion in *F. tularensis*. Moreover, in contrast to Barker *et al*. who failed to demonstrate secretion of PdpE-CyaA [35], a PdpE-TEM fusion was secreted in our hands during LVS as well as *F. novicida* U112 infections. The discrepancy between the results obtained using the CyaA and TEM assays suggests that the different types of reporter fusions may have adverse effects on secretion, however, why the CyaA method indicated that secretion of VgrG was FPI-independent is unclear. Importantly, while the TEM-tag in several instances was found to interfere with FPI protein function in terms of its ability to support intracellular growth and/or LDH release of the corresponding LVS mutant, it did not have a general impact on the ability of the protein to be secreted as 4 out of 5 identified substrates tested in these additional assays (*i.e.* fusions of IglE, IglC, VgrG and IglF) failed to promote growth but were still secreted. In contrast to the previous studies that investigated secretion of FPI proteins (above), the present analysis was much more comprehensive and included all of the 17 conserved FPI proteins. The data corroborated previous findings that VgrG and IglI were secreted, but also demonstrated, somewhat more unexpectedly, that secretion of IglE, IglC, PdpE, PdpA, IglJ and IglF also occurred. The findings indicate that the *F. tularensis* T6SS possesses unique substrates since all proteins except for VgrG are unique to the bacterium, although IglC was recently suggested to be a remote homologue of Hcp [42]. A majority of the identified substrates are predicted to have an unknown location within the bacterium, with the exception of PdpA, which according to PsortB may be an outer membrane (OM) protein. From empirical studies, it has been shown that PdpE as well as IglE are OM proteins, while IglC is a soluble protein, and IglI exist in both soluble and membrane fractions [32], [52], [53]. Only IglE has been suggested to contain a trans-membrane region, which according to TMHMM resides within its N-terminus and thus overlaps the putative signal peptide (below). While the eight proteins do not share any obvious common traits, five of them, IglE, IglC, VgrG, PdpE, and IglJ, are among the smallest of the FPI proteins. Interestingly, a very comprehensive study on the *Legionella* T4SS suggested that among 164 translocated proteins, there was a clear bias for small substrates. Thus, it is possible that it is beneficial for the bacteria to secrete smaller substrates since it most likely is more energy-efficient. Our findings based on the use of the PMF-inhibitor CCCP demonstrated that the PMF appears to play a very important role for generating the energy required for T6S in *Francisella*, which is in agreement with its critical role for other forms of protein export, *e.g.*, that related to the T3SS and flagellar assembly [45], [46], [47], [48]. The general belief is that substrate export via the T6SS is thought to occur in a Sec-independent fashion, as a one-step mechanism across both bacterial membranes [26], however, according to SignalP, both PdpE and IglE may possess N-terminal signal peptides. Since a functional T6SS was shown to be essential for their export during an LVS infection, this raises the question of whether Sec and T6SS may be connected. A precedent for this comes from a recent study on Burkholderia *cenocepacia*, where the T6SS was shown to mediate the disruption of the membranes of bacteria-containing vacuoles, which then allowed the escape of proteins secreted by the Type II secretion system (T2SS) into the macrophages cytoplasm [54].

The bulk of the data was based on the 18 h time point to minimize the issue with low detection levels, since bacterial numbers are high at this time point and host cell lysis still has not occurred. Also, from a kinetic experiment, secretion was shown to be significantly lower or non-detectable at earlier time points (9 h and even more so at 3 h) for all substrates tested. Still, our data do not rule out that FPI proteins are secreted early during the phagosomal stage, however, the sensitivity of the TEM assay may not be sufficient to detect secretion at this stage. A caveat when comparing the levels of secretion of substrates is that the levels of the proteins will affect the secreted amounts. To this end, we attempted to equilibrate the expression levels by expressing all proteins under the same, strong promoter. In addition, we determined the actual levels by Western blot analysis of bacterial lysates and observed expression of most proteins, with the exception of DotU, PdpB and PdpC, which all appeared unstable when fused to TEM. Thus, this suggests that our quantification likely reflects the efficiency of the secretion machinery. Our data do not explain why the IglC and IglE proteins are more effectively secreted, but a simple explanation could be that the higher export merely reflects the need for larger quantities of these proteins to establish their essential function outside of the bacterium. For example, one could envisage that the formation of dynamic Hcp-like tubular structures on the bacterial surface may require large amounts of secreted IglC. In T3SSs, a sorting platform that promotes a secretion hierarchy among the secreted substrates has been described [55]. The sequential loading of this platform, facilitated by customized chaperones and their affinity to the platform, ensures the hierarchy in protein secretion [55]. While chaperones so far are unknown to T6SSs, the existence of mechanisms that favor the secretion of one substrate over another still cannot be ruled out.

Very few studies of T6SSs have involved a comprehensive identification of the secreted substrates to date. One notable exception is the study by Russell *et al*. where mass spectrometry was used to identify secretion of T6SS-dependent substrates by *B. thailandensis* grown in broth. In total, 11 proteins were considered to be putative T6S-substrates. In contrast, our findings are based on an intracellular pathogen that appears to very tightly regulate its T6SS-dependent secretion and may require signals unique to the intracellular environment to become activated. While there appears to be no similarities between the substrates identified in the two studies, the possibility that *F. tularensis* may encode T6S substrates outside of the FPI cannot be dismissed. Nevertheless, the substrates are likely to serve very different purposes since it was hypothesized that several of the *Burkholderia* substrates contributed to inter-bacterial competition, whereas all the evidence regarding the FPI proteins indicate that their critical roles are to modify the intracellular habitat to make it permissive for replication of *F. tularensis*.

Our data do not identify if the substrates also perform effector functions within the host cell. Nevertheless, even simply as part of the surface-located structure of the T6S machinery, they still could contribute to the effector function of the T6SS by directly interacting with the effectors. Precedence for this hypothesis has been provided by the demonstration of a direct interaction between the secreted effector protein EvpP and Hcp in the fish pathogen *Edwardsiella tarda* [25]. In addition, a bioinformatic analysis of *Pantoea* and *Erwinia*, which harbor up to four T6SS loci, have shown that each of them contain distinct VgrG proteins that appear to have different phylogenetic origins than the other conserved parts of the T6SS loci [21]. Since these VgrG variants in many instances contain C-terminal domains that are homologous to regions of putative T6SS effector proteins, it has been suggested that orphan VgrG proteins without C-terminal extensions may physically interact with and carry similar effector proteins through the secretion machinery, in the same way as was suggested for *Edwardsiella* Hcp. Such VgrG- and Hcp-effector combinations may perform distinct biological functions; thereby contributing to the extensive functional diversification that appears to be a hallmark of T6SSs. Analogously, the identified *F. tularensis* substrates could contribute to the diversity of effector mechanisms that have been linked to the *F. tularensis* T6SS by such an interaction, even if they are not effectors *per se*.

The present results indicate that the background observed with the method was minimal since less than 0.5% of the cells infected with LVS expressing any of the other TEM constructs displayed positive signals, *i.e.* fluoresced blue. They also demonstrate that bacterial lysis was not an issue at the time points tested, since this could otherwise have resulted in the unspecific delivery of TEM fusions to the host cytosol, thereby generating false positives. Moreover, the lack of any noticeable secretion in the Δ*dotU*, Δ*vgrG* and Δ*iglC* backgrounds further corroborates the utility of the method. However, one drawback of using these three mutants is they lack intracellular replication, thus, the number of bacteria present will be limited and, therefore, possibly secretion may be more difficult to detect. To that end, we also investigated secretion from the Δ*iglG* mutant since it replicates as well as LVS in J774 cells, however, also this mutant background led to essentially abolished secretion for most of the substrates tested. Therefore, the data clearly suggest that the FPI constitutes a secretion system and that each of the DotU, VgrG, IglC and IglG proteins represent functional components necessary for secretion.

Another potential caveat with the experimental approach is that the quantification was performed with the LVS strain, and not a FPI mutant, since we wanted to ensure that the bacterial numbers were similar and thereby did not bias the amounts of secreted proteins. This could mean that the efficiency of the secreted fusion proteins was affected by competition with the native, non-tagged FPI proteins. This may be one explanation as to why, although the levels of cells positive for TEM secretion were higher than background levels, they still did not represent a majority of the infected cells although the infection protocol used here routinely leads to infection frequencies of >95% [56]. A recent TEM-study on the *Coxiella burnetii* T4SS revealed substrates with distinct secretion efficiencies that ranged from 1 to 90% when wild-type *Coxiella* was used to infect U937 cells [39]. Therefore, we believe that the limited secretion that we observe upon infection with *Francisella* may indeed be accurate, rather than stem from a technical problem with the secretion assay.

In contrast to the utility of the TEM assay for LVS, we could not apply it directly on the *F. novicida* U112 strain, since it showed very intense secretion of the native β-lactamase encoded by *FTN_1072*. When expressed *in trans* in an *FTN_1072* mutant of *F. novicida* or in LVS, the enzyme was secreted, although more efficiently in the former strain. To determine whether secretion in general is more efficient in *F. novicida*, we introduced TEM-fusions of IglE, IglC, VgrG, IglI, PdpE, PdpA, IglJ and IglF into *FTN_1072*. Surprisingly, only PdpE, IglE, IglC and, to a minor extent also PdpA, were secreted using the identical set-up as for LVS. This suggests that PdpA, PdpE, IglE and IglC are common substrates of the T6SS of *Francisella* spp, but also that there must be fundamental differences in T6S and/or regulation thereof in the aforementioned species. Surprisingly, among the eight proteins, PdpE is one of the least conserved substrate, with a total of 21 amino acid substitutions across the entire protein and the *novicida* variant is three residues shorter. IglJ and IglF also exhibit sequence variation between the species, with a total of 23 and 24 substitutions respectively, and the *F. novicida* IglF homologue being 22 residues longer. In contrast, IglC is identical between the two species, while VgrG, IglE and IglI only contain 1, 2, and 8 substitutions respectively. Thus, sequence diversity is not a likely explanation as to why there were distinct secretion patterns in the two subspecies. Studies on other non-*Francisella* T6SSs have revealed that secretion appears to be tightly regulated and at times difficult to detect in the experimental systems, likely indicating that that they are upregulated only under specific situations *in vivo*. For example, detection of Hcp and VgrG secretion in *P. aeruginosa* required the introduction of a chromosomal mutation in *retS* [57], [58], and in the case of *V. cholerae* 01, secretion has only been detected under certain temperatures and osmolarity [59]. The same is likely relevant also for *F. tularensis*, in fact, the first described FPI protein, IglC, was initially identified as a protein upregulated during intramacrophage infection [60]. A transcriptional analysis has revealed that many FPI genes are upregulated during two phases of the *F. tularensis* intracellular infection, with the maximum mRNA levels observed at 12–16 h, *i.e.*, at the end of the cytosolic replication stage [61]. These results also corroborate ours, demonstrating that secretion of FPI substrates increases from 9 h to 18 h. Moreover, we also have data that suggest that the FPI proteins IglA and IglC play an additional role post-phagosomal escape, as only LVS bacteria, but not Δ*iglA* or Δ*iglC* mutant bacteria, will grow upon microinjection into the cytosol of J774 cells (Meyer, Bröms and Sjöstedt, unpublished).

The T6SS of *F. tularensis* appears to be distinct from a bioinformatic standpoint, and in the present study we have shown that these differences also extend to the secreted substrates, as most of those identified lack apparent homologues in other bacterial systems. A reason for this may be that most of the prototypically T6SS studied so far are harbored by enterobacteria with an extracellular life style, which contrasts to the intracellular life style of *F. tularensis*. Instead, the FPI may represent an adaptation of a T6SS to the macrophage habitat and/or originally to alternative intracellular habitats of the bacterium such as amoeba. Future functional analysis of the substrates identified in this study, where putative effector protein(s) will be distinguished from those that constitute structural components, should provide molecular mechanisms that account for the unique intracellular life cycle of this important pathogen.

## Materials and Methods

### Bacterial strains, plasmids and growth conditions

Bacterial strains and plasmids used in this study are listed in Table S1. *Escherichia coli* strains were cultured in Luria Bertani broth (LB) or on Luria agar plates at 37°C. *F. tularensis* was grown on modified GC-agar base (Difco GC medium base [Becton Dickinson] complemented with hemoglobin and Iso-Vitalex) at 37°C. When necessary, tetracycline (10 µg/ml for *E. coli*, 5 µg/ml for *F. tularensis* or *F. novicida*), or kanamycin (50 µg/ml for *E. coli*, 10 µg/ml for *F. tularensis* or *F. novicida*) were used. For *in vitro* growth experiments of *F. tularensis*, LVS was grown over night in Chamberlain's medium [62] at 37°C, 200 rpm with good aeration. Next day, bacteria were subcultured to OD_600_  = 0.15 and grown for an additional 24 h, during which OD_600_ was measured at different time points. For CCCP stress experiments, the substance was added at a final concentration of 0, 1 or 10 µM to the subcultures when they had reached OD_600_  = 0.4. To determine whether the effects of the substance were reversible, bacteria were pelleted after 3 h, washed once in PBS and redissolved in Chamberlain's medium lacking CCCP.

### Construction of TEM expression vectors

Plasmids used in this study are listed in Table S1. Primer combinations and restriction enzymes used to generate the plasmids are listed in Table S2. All fragments were amplified by the Expand Long Range dNTPack (Roche) and were initially cloned into the pCR4-TOPO TA cloning vector (Invitrogen) to facilitate sequencing. LVS chromosomal DNA was commonly used as template in the PCR reactions with the following exceptions: When *dotU*, *icmF* and *iglJ* were cloned by *Nde*I*-*digestion, the templates used were *dotU*, *icmF* [34] and *iglJ* alleles engineered by overlap PCR to lack their intrinsic *Nde*I-sites. Since PdpD is significantly truncated by an in-frame stop codon in LVS, we used *F. tularensis* subsp. *novicida* U112 as template in the overlap PCR reaction to amplify full-length *pdpD* without its intrinsic *Nde*I site. The expression vector pJEB709 encoding for the mature TEM β-lactamase from *E. coli* was constructed by PCR amplification of the TEM coding sequence from plasmid pCX340 [36] using primers TEM_F and TEM_R, and introducing the resulting fragment into the *Kpn*I-*Eco*RI sites of pMOL42 [34]. Next, PCR-amplified FPI genes lacking their native stop codons were introduced as *Nde*I-*Kpn*I fragments into pJEB709, to generate translational C-terminal TEM fusion proteins under the control of the constitutive *groE* promoter. To express the fusion proteins in Km-resistant clones of *F. novicida*, the encoding gene fusions were generally excised as *Nde*I-*Eco*RI fragments from pJEB709 and introduced into pKK214 [63]. Genes *FTN_1072* and *FTL_0879* encoding native *Francisella* β-lactamases were also amplified as *Nde*I-*Eco*RI fragments and introduced into this vector. Since *pdpA* possesses an intrinsic *Eco*RI-site, we instead lifted it by *Nde*I-*Kpn*I digestion from the TA cloning vector into pKK214 that originally encoded PdpE-TEM, but from which the intrinsic *pdpE* gene had first been excised using *Nde*I-*Kpn*I digestion. All plasmids were transferred into *F. tularensis* or *F. novicida* by electroporation.

### Western blot analysis

Bacterial lysates were prepared in Laemmli sample buffer and boiled prior to separation on 10% sodium dodecyl sulfate (SDS)-polyacrylamide gels. Proteins were transferred onto nitrocellulose membranes using a semidry blotter (Bio-Rad laboratories, CA, USA). Membranes were probed with mouse monoclonal antibodies against TEM β-lactamase (Abcam, Cambridge, MA) or against IglB (BEI Resources, Manassas, VA, USA), followed by a secondary goat anti-mouse antibody (Santa Cruz Biotechnology, CA, USA), For detection, the Enhanced Chemiluminescence system (ECL) (Amersham Biosciences, Uppsala, Sweden) was used.

### Cultivation of macrophages and the TEM secretion assay

J774A.1 macrophages (ATCC TIB-67) were used throughout this study, cultured and maintained in DMEM (GIBCO BRL, Grand Island, NY, USA) with 10% heat-inactivated FBS (GIBCO). The day before infection, macrophages were seeded onto BD Falcon 8-wells glass chambers slides (BD Biosciences, Bedford, MA, USA) in fresh culture medium at 1.5 x 10^5^ cells/well. Following incubation overnight, cells were washed, reconstituted with fresh culture medium and allowed to recover for at least 30 min. After 2 h of infection using a multiplicity of infection (MOI) of 200, the cells were washed three times and incubated in fresh medium containing 5 µg/ml gentamicin (equals time point 0 h). These conditions resulted in 1–3 bacteria/infected cell and approximately 80–100% infected cells. To study the effects of inhibitors, the medium was at this time point supplemented with 1 or 10 µM of the proton motive force inhibitor Carbonyl cyanide *m*-chlorophenylhydrazone (CCCP) (Sigma, MO, USA), which had been freshly dissolved in DMSO. To control for possible adverse effects, DMSO was also added to controls not treated with substances. In all cases, the final concentration of DMSO in cultures was less than 0.05%. At 3 h, 9 h, 18 h or 24 h, cells were washed twice with PBS before loading them with CCF2-AM according to the manufacturer's instructions (Invitrogen, CA, USA). To prevent export of the CCF2 substrate, cells were loaded at RT and Probenicid (Invitrogen) was added at a final concentration of 2.5 mM to the loading mixture. Translocation of β-lactamase fusions into CCF2-loaded cells was determined after 60–90 min of incubation, by counting the number of blue fluorescent cells in images taken with a live-cell imaging microscope (Nikon Eclipse Ti-E) equipped with a Nikon DS-U2/L2 camera, using a Chroma beta-lactamase double filter. Loaded cells that did not exhibit secretion of TEM fusions appeared green. Images were assembled using Adobe Photoshop CS2 (Adobe Systems, San Jose, CA). Each strain was tested on at least 4 separate occasions using duplicate wells. For statistical analysis of blue *vs* green fluorescent cells, an average of 4,000–8,000 cells that included pictures from all separate experiments for a particular strain was counted. Each picture was considered an independent observation and used to calculate the average% of blue fluorescent cells and the standard error of the mean (SEM) for a particular strain. For CCCP experiments, an average of 40,000–60,000 cells from a total of 6 separate experiments was counted. A TEM-fusion was considered to be non-secreted if it resulted in less than 1 blue fluorescent cell per 200 cells, *i.e.* the cut-off of the assay was<0.5%. For infections where only green cells were observed, for statistical purposes we assumed that the percentage of blue fluorescent cells was half the cut-off value, *i.e.* 0.25%. Student‚s t-test was used to determine whether the% of blue fluorescent cells differed significantly between parental strains and strains harboring FPI-TEM fusions, or whether different concentrations of CCCP had any effect on protein secretion.

### Intracellular replication in macrophages

To determine the ability of *F. tularensis* to grow within macrophages, J774A.1 cells were infected for 2 h using an MOI of 200, washed three times, and incubated in the presence of 5 µg/ml gentamicin for 30 min (corresponds to time zero). When appropriate, the medium was supplemented with 1 or 10 µM CCCP onwards. At indicated time points, the macrophage monolayers were lysed in PBS with 0.1% deoxycholate, serially diluted in PBS and plated on modified GC-agar base plates for determination of viable counts. A two-sided *t*-test with equal variance was used to determine whether the growth of a strain differed significantly from that of LVS (mutant complementation study) or non-treated LVS (CCCP study).

### LDH release assay

J774A.1 cells were infected as described in “Intracellular replication in macrophages” and supernatants were sampled at different time points and assayed for the presence of released Lactate dehydrogenase (LDH) using the CytoTox 96 Non-radioactive cytotoxicity assay (Promega, Madison, USA). Data are means ± standard deviations of three wells from one representative experiment of three. Uninfected cells lysed in PBS with 0.1% deoxycholate served as a positive control, and the value for this control was arbitrarily considered 100% cell lysis. Sample absorbance was expressed as the percentage of the positive control value. A two-sided *t*-test with equal variance was used to determine whether the cytopathogenicity of a strain differed significantly from that of LVS (mutant complementation study) or non-treated LVS (CCCP study).

## Supporting Information

Figure S1 ***In vitro*** **growth of** ***F. tularensis*** **in the presence of CCCP.** LVS grown over night in Chamberlain's medium at 37°C, was subcultured to OD_600_  = 0.15 and grown for an additional 24 h, during which OD_600_ was measured at different time points. The PMF inhibitor CCCP was added at a final concentration of 0, 1 or 10 µM to the subcultures when they had reached OD_600_  = 0.4.
(TIF)

Figure S2 **Intracellular growth (A) and cytopathogenicity (B) of LVS.** (A) J774 cells were infected by LVS at an MOI of 200 for 2 h. Upon gentamicin treatment, cells were allowed to recover for 30 min after which they were lysed immediately (corresponds to 0 h; light gray bars) or after 9 h (dark gray bars) or 18 h (black bars) with PBS-buffered 0.1% sodium deoxycholate solution and plated to determine the number of viable bacteria (log_10_). All infections were repeated two times and a representative experiment is shown. Each bar represents the mean values and the error bar indicates the standard deviation from triplicate data sets. The asterisk indicates that the log_10_ number of CFU recovered from CCCP treated cells was significantly different at a given time point as determined by a 2-sided *t*-test with equal variance (*, *P*≤0.05). (B) Culture supernatants of LVS-infected or uninfected J774 cells were assayed for LDH activity at 0, 9 and 18 h post infection and the activity was expressed as a percentage of the level of non-infected lysed cells (positive lysis control). Shown are means and standard deviations of triplicate wells from one representative experiment of two. The asterisks indicate that the cytopathogenicity levels were significantly different for CCCP treated cells at a given time point as determined by a 2-sided *t*-test with equal variance (*, *P*≤0.05; ***, *P*≤0.001).
(TIF)

Table S1 **Strains and plasmids used in this study.**
(DOC)

Table S2 **Oligonucleotides used in this study.**
(DOC)
