# Supplementary figures and images for: Unique Substrates Secreted by the Type VI Secretion System of Francisella tularensis during Intramacrophage Infection
Source: PLoS One. 2012 Nov 20;7(11):e50473. doi: 10.1371/journal.pone.0050473 (PMC3502320; doi:10.1371/journal.pone.0050473)

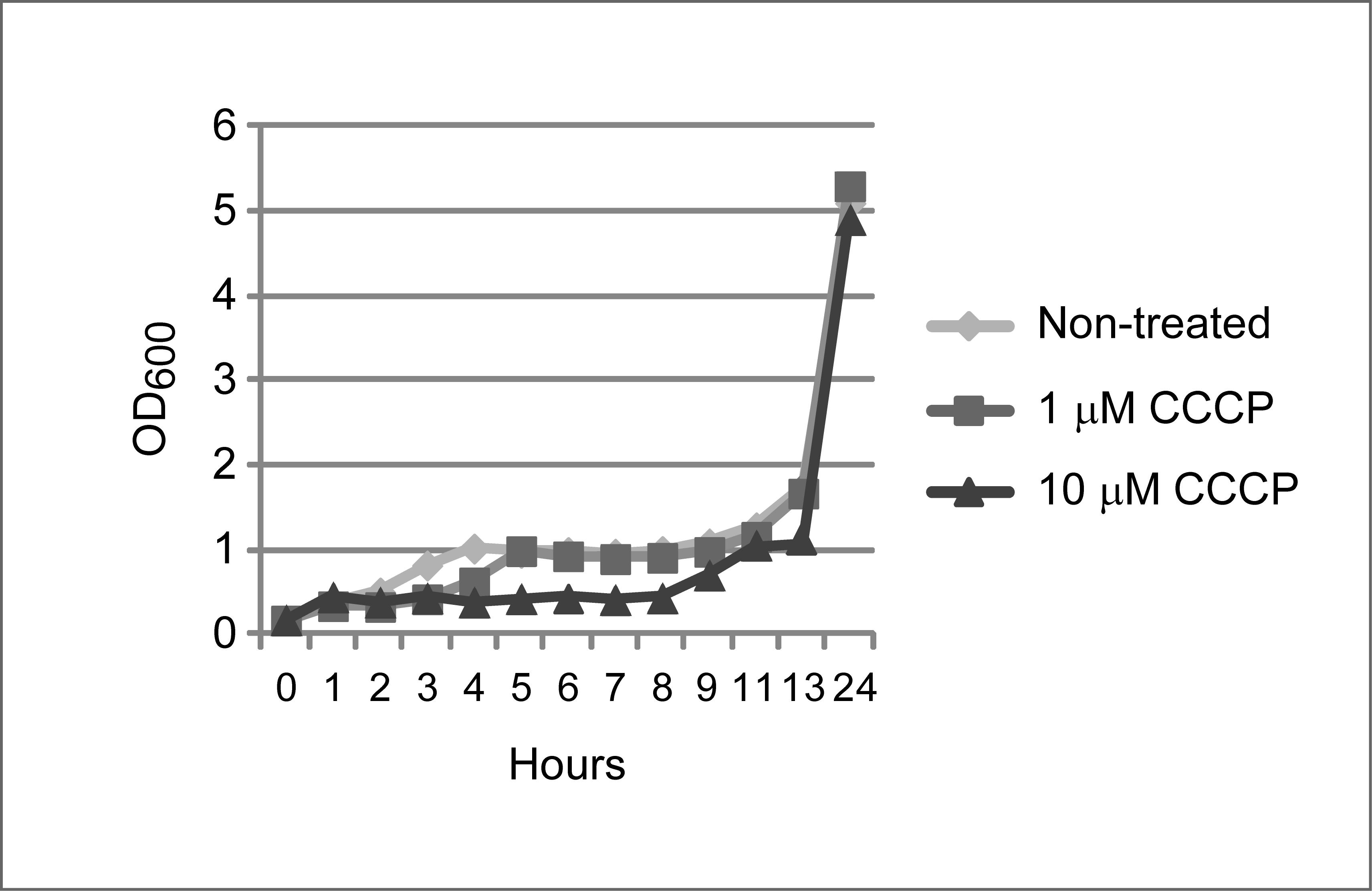

Supplement: Figure S1 — In vitro growth of F. tularensis in the presence of CCCP. LVS grown over night in Chamberlain's medium at 37°C, was subcultured to OD600 = 0.15 and grown for an additional 24 h, during which OD600 was measured at different time points. The PMF inhibitor CCCP was added at a final concentration of 0, 1 or 10 µM to the subcultures when they had reached OD600 = 0.4. (TIF) [file pone.0050473.s001.tif]

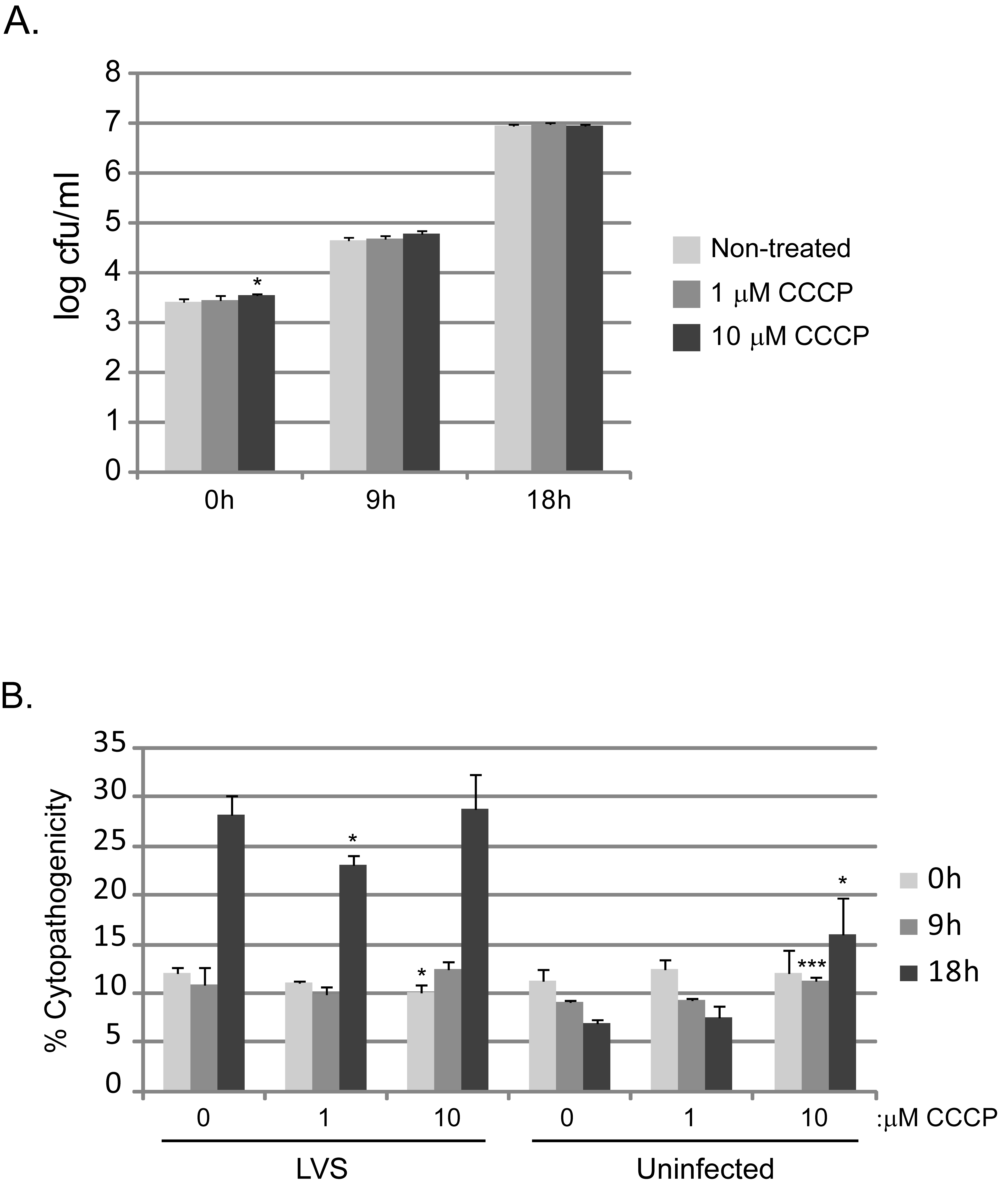

Supplement: Figure S2 — Intracellular growth (A) and cytopathogenicity (B) of LVS. (A) J774 cells were infected by LVS at an MOI of 200 for 2 h. Upon gentamicin treatment, cells were allowed to recover for 30 min after which they were lysed immediately (corresponds to 0 h; light gray bars) or after 9 h (dark gray bars) or 18 h (black bars) with PBS-buffered 0.1% sodium deoxycholate solution and plated to determine the number of viable bacteria (log10). All infections were repeated two times and a representative experiment is shown. Each bar represents the mean values and the error bar indicates the standard deviation from triplicate data sets. The asterisk indicates that the log10 number of CFU recovered from CCCP treated cells was significantly different at a given time point as determined by a 2-sided t-test with equal variance (*, P≤0.05). (B) Culture supernatants of LVS-infected or uninfected J774 cells were assayed for LDH activity at 0, 9 and 18 h post infection and the activity was expressed as a percentage of the level of non-infected lysed cells (positive lysis control). Shown are means and standard deviations of triplicate wells from one representative experiment of two. The asterisks indicate that the cytopathogenicity levels were significantly different for CCCP treated cells at a given time point as determined by a 2-sided t-test with equal variance (*, P≤0.05; ***, P≤0.001). (TIF) [file pone.0050473.s002.tif]
