# Supplementary material for: Unique Substrates Secreted by the Type VI Secretion System of Francisella tularensis during Intramacrophage Infection
Source: PLoS One. 2012 Nov 20;7(11):e50473. doi: 10.1371/journal.pone.0050473 (PMC3502320; doi:10.1371/journal.pone.0050473)
Supplement: Table S1 — Strains and plasmids used in this study. (DOC) [file pone.0050473.s003.doc]

**Table S1. Strains and plasmids used in this study**

| Strain or plasmid | Relevant genotype or phenotype | Source or reference |
| --- | --- | --- |
| Strain |  |  |
| *E. coli* |  |  |
| TOP10 | F-*mcrA*, Δ(*mrr*-*hsdRMS-mcrBC*), φ80*lacZ*ΔM15, Δ*lacX*74, *recA1*, *deoR*, *araD*139, Δ(*ara-leu*)7679, *galU*, *galK*, *rpsL* (Str^R^), *endA1*, *nupG* | Invitrogen |
| *F. tularensis* |  |  |
| LVS | Live vaccine strain | USAMRIID^1^ |
| Δ*vgrG* | LVS, *vgrG* in-frame deletion mutant of codons 4-162 |  |
| Δ*iglG* | LVS, *iglG* in-frame deletion of codons 3-169 |  |
| Δ*dotU* | LVS, *dotU* in-frame deletion mutant of codons 4-203 |  |
| Δ*iglC* | LVS, *iglC* in-frame deletion mutant of codons 28-205 |  |
| U112 | *F. novicida*, wild-type |  |
| *FTN_1072* | *F. novicida*, *FTN_1072* insertion mutant, Km^R^ |  |
| *FTN_1002* | *F. novicida*, *FTN_1002* insertion mutant, Km^R^ |  |
| Plasmid |  |  |
| pCR®4-TOPO® | TA cloning vector, Km^R^, Cb^R^ | Invitrogen |
| pCX340 | Cloning vector used to construct TEM β-lactamase fusion proteins, Tet^R^ |  |
| pMOL42 | pKK289Km carrying pUC19 MCS from *Hind*III to *Eco*RI, with an upstream *Nde*I site, Km^R^ |  |
| pJEB709 | pMOL42 derivative encoding mature TEM β-lactamase from *E. coli*, Km^R^ | This study |
| pSK001 | pJEB709, encoding PdpA-TEM, Km^R^ | This study |
| pSK002 | pJEB709, encoding IcmF-TEM, Km^R^ | This study |
| pSK003 | pJEB709, encoding IglE-TEM, Km^R^ | This study |
| pMOL95 | pJEB709, encoding VgrG-TEM, Km^R^ | This study |
| pSK004 | pJEB709, encoding IglF-TEM, Km^R^ | This study |
| pMOL140 | pJEB709, encoding IglG-TEM, Km^R^ | This study |
| pMOL91 | pJEB709, encoding IglH-TEM, Km^R^ | This study |
| pSK005 | pJEB709, encoding DotU-TEM, Km^R^ | This study |
| pMOL138 | pJEB709, encoding IglI-TEM, Km^R^ | This study |
| pSK006 | pJEB709, encoding IglJ-TEM, Km^R^ | This study |
| pSK007 | pJEB709, encoding PdpC-TEM, Km^R^ | This study |
| pMOL94 | pJEB709, encoding PdpE-TEM, Km^R^ | This study |
| pJEB724 | pJEB709, encoding IglD-TEM, Km^R^ | This study |
| pJEB733 | pJEB709, encoding IglC-TEM, Km^R^ | This study |
| pJEB732 | pJEB709, encoding IglB-TEM, Km^R^ | This study |
| pJEB726 | pJEB709, encoding IglA-TEM, Km^R^ | This study |
| pSK008 | pJEB709, encoding PdpD-TEM, Km^R^ | This study |
| pKK214 | Expression vector containing the *groE* promoter, Tet^R^ |  |
| pJEB932 | pKK214, encoding PdpA-TEM, Tet^R^ | This study |
| pSK009 | pKK214, encoding IglE-TEM, Tet^R^ | This study |
| pMOL147 | pKK214, encoding VgrG-TEM1, Tet^R^ | This study |
| pJEB935 | pKK214, encoding IglF-TEM1, Tet^R^ | This study |
| pJEB930 | pKK214, encoding IglG-TEM, Tet^R^ | This study |
| pMOL145 | pKK214, encoding IglI-TEM1, Tet^R^ | This study |
| pJEB936 | pKK214, encoding IglJ-TEM1, Tet^R^ | This study |
| pMOL148 | pKK214, encoding PdpE-TEM, Tet^R^ | This study |
| pMOL146 | pKK214, encoding IglC-TEM1, Tet^R^ | This study |
| pSK010 | pKK214, encoding FTN_1072, Tet^R^ | This study |
| pJEB931 | pKK214, encoding FTL_0879, Tet^R^ | This study |

^1^ US Army Medical Research Institute of Infectious Diseases, Fort Detrick, Frederick, MD

**References**

1. Bröms JE, Meyer L, Lavander M, Larsson P, Sjöstedt A (2012) DotU and VgrG, core components of type VI secretion systems, are essential for *Francisella* LVS pathogenicity. PLoS One 7: e34639.

2. Bröms JE, Lavander M, Meyer L, Sjöstedt A (2011) IglG and IglI of the *Francisella* pathogenicity island are important virulence determinants of *Francisella tularensis* LVS. Infect Immun 79: 3683-3696.

3. Golovliov I, Sjöstedt A, Mokrievich A, Pavlov V (2003) A method for allelic replacement in *Francisella tularensis*. FEMS Microbiol Lett 222: 273-280.

4. Anthony LD, Burke RD, Nano FE (1991) Growth of *Francisella* spp. in rodent macrophages. Infect Immun 59: 3291-3296.

5. Gallagher LA, Ramage E, Jacobs MA, Kaul R, Brittnacher M, et al. (2007) A comprehensive transposon mutant library of *Francisella novicida*, a bioweapon surrogate. Proc Natl Acad Sci U S A 104: 1009-1014.

6. Charpentier X, Oswald E (2004) Identification of the secretion and translocation domain of the enteropathogenic and enterohemorrhagic *Escherichia coli* effector Cif, using TEM-1 beta-lactamase as a new fluorescence-based reporter. J Bacteriol 186: 5486-5495.

7. Bönquist L, Lindgren H, Golovliov I, Guina T, Sjöstedt A (2008) MglA and Igl proteins contribute to the modulation of *Francisella tularensis* live vaccine strain-containing phagosomes in murine macrophages. Infect Immun 76: 3502-3510.

8. Kuoppa K, Forsberg Å, Norqvist A (2001) Construction of a reporter plasmid for screening *in* *vivo* promoter activity in *Francisella tularensis*. FEMS Microbiol Lett 205: 77-81.
