# Supplementary material for: Unique Substrates Secreted by the Type VI Secretion System of Francisella tularensis during Intramacrophage Infection
Source: PLoS One. 2012 Nov 20;7(11):e50473. doi: 10.1371/journal.pone.0050473 (PMC3502320; doi:10.1371/journal.pone.0050473)
Supplement: Table S2 — Oligonucleotides used in this study. (DOC) [file pone.0050473.s004.doc]

Table S2. Oligonucleotides used in this study

| Insert (Vector ) | Oligonucleotide pair(s) | |
| --- | --- | --- |
| TEM (pJEB709) | | TEM_F: 5´-*GGT ACC* CAC CCA GAA ACG CTG GTG A-3´ (*Kpn*I) and TEM_R: 5´-*GAA TTC* TTA CCA ATG CTT AAT CAG TGA G-3´ (*Eco*RI) |
| PdpA-TEM (pSK001, pJEB932) | | Y2H_pdpA1F: 5´-*CAT ATG* ATA GCA GTA AAA GAT ATA ACT GAT-3´ (*Nde*I) and PdpA_KpnI_R: 5´-*GGT ACC* ATT TCC TTT TGA TTT ATA TCT TAG-3´ (*Kpn*I) |
| IcmF-TEM (pSK002) | | PdpB_NdeI_F: 5´-*CAT ATG* AAT TTT ATT AAA AAT CAT CAA ATA TT-3´ (*Nde*I) and PdpB_KpnI_R: 5´-*GGT ACC* TTG TAC ATT GAC TTC TCC TTG T-3´ (*Kpn*I) |
| IglE-TEM (pSK003, pSK009) | | pigA_for: 5´-*CAT ATG* TAC AAT AAA TTA TTG AAA AAT C-3´ (*Nde*I) and pigA_gsk_rev: 5´-*GGT ACC* atc ttt ttc tat gct act atc-3´ (*Kpn*I) |
| VgrG-TEM (pMOL95, pMOL147) | | PigB_F: 5´-*CAT ATG* TCA AAA GCA GAC CAT ATT T-3´ (*Nde*I) and PigB_GSKrev: 5´-*GGT ACC* TCC AAC CAT TGT TGC TGT AG- 3´ (*Kpn*I) |
| IglF-TEM (pSK005; pJEB935) | | pigC_for: 5´-*CAT ATG* AAT AAT AAT ATT GAT AAA TGG TTT GA-3´ (*Nde*I) and pigC_gsk_rev: 5´-*GGT ACC* AGC ACC AAA AAA AAA ACT ATT TG-3´ (*Kpn*I) |
| IglG-TEM (pMOL140, pJEB930) | | pigD_for: 5´-*CAT ATG* TTA AAT ATT ATA AAT GAC TCC-3´ (*Nde*I) and pigD_gsk_rev: 5´-*GGT ACC* AGA TGT TTT TAC ATT TAT TTG TC-3´ |
| IglH-TEM (pMOL91) | | pigE_for: 5´-*CAT ATG* GAT GAA AAA AGA AAA GAT TTA AGT A-3´ and pigE_gsk_rev: 5´-*GGT ACC* TAT AGA GTT ATT TAA AAC AAT CTT T-3´ |
| DotU-TEM (pSK005) | | DotU_NdeI_F :5´-*CAT ATG* AAA GAC TTT AAA GAG ATA GAA ATT-3´ (*Nde*I) and PigF_GSKrev: 5´-*GGT ACC* CCA GCT TAA TAA AAT TAG TAA G-3´ (*Kpn*I) |
| IglI-TEM (pMOL138, pMOL145) | | pigG_for: 5´-*CAT ATG* AGT CAG ATA ATA TCT ACA C-3´ (*Nde*I) and pigG_gsk_rev: 5´-*GGT ACC* TAT GTC AAA AAG ATC TTC AAA ATA-3´ (*Kpn*I) |
| IglJ-TEM (pSK006; pJEB936) | | IglJ_NdeI_F: 5´-*CAT ATG* AAG ACT ATT TTG AAG ATC TT-3´ (*NdeI*) and IglJ_mut_rev: 5´-A**G**A AGG AAT ATA TGC CCC CAA-3´ IglJ_mut_F: 5´-GCA TAT ATT CCT T**C**T TAT GTT TAT ATT ATA AAC ATT AAG and IglJ_KpnI_R: 5´-*GGT ACC* TAA ATT AAA ATA ACT TAG GTA TAT CTG-3´ (*Kpn*I) |
| PdpC-TEM (pSK007) | | PdpC_NdeI_F: 5´-*CAT ATG* AAC GAC AAA TAT GAA CTA AAT-3´ (*NdeI*) and PdpC_KpnI_R: 5´-GGT ACC TGA TGA TAT TTT TTT AAA AAA GTC TGA-3´ (*Kpn*I) |
| PdpE-TEM (pMOL94, pMOL148) | | pigI_for: 5´-*CAT ATG* AGT AAA AAA ATA TTT AAA TTA TTA-3´ (*Nde*I) and pigI_gsk_rev: 5´-*GGT ACC* TAT TAT AGT AAT TTT CTT TTC ATA AT-3´ (*Kpn*I) |
| IglD-TEM (pJEB724) | | IglD_Y2H_F: 5´-*CAT ATG* TTT CTA GAA AGG ATT TAT TGG GAA GAT-3´ (*Nde*I) and IglD_KpnI_R: 5´-*GGT ACC* AGA AAA GGC TAT AAA GAA ATC AA-3´ (*Kpn*I) |
| IglC-TEM (pJEB733, pMOL146) | | IglC_GSK_F: 5´-*CAT ATG* AGT GAG ATG ATA ACA AGA CAA CAG GTA-3´ (*Nde*I) and IglC_KpnI_R: 5´-*GGT ACC* TGC AGC TGC AAT ATA TCC TAT-3´ (*Kpn*I) |
| IglB-TEM (pJEB732) | | IglB_Y2H_F: 5´-*CAT ATG* ACA ATA AAT AAA TTA AGT CTC ACT GAT G-3´ (*Nde*I) and IglB_KpnI_R: 5´- *GGT ACC* GTT ATT ATT TGT ACC GAA TAA TTC-3´ (*Kpn*I) |
| IglA-TEM (pJEB726) | | IglA_GSK_F: 5´-CAT ATG GCA AAA AAT AAA ATC CCA AAT TCA AGG-3´ (NdeI) and IglA_KpnI_R: 5´-GGT ACC CTT ACC ATC TAC TTG TTG ATT A-3´ (KpnI) |
| PdpD-TEM (pSK008) | | PdpD_NdeI_F: 5´-*CAT ATG* GAT CAA GAT ATC AAC GAT TTA TTA T-3´ (*Nde*I) and PdpD_NdeImut_R: 5´-AAA CAT **G**TG TCT TTC AAC GTC AT-3´  PdpD_NdeImut_F: 5´-TGA CGT TGA AAG ACA **C**AT GTT T-3´ and PdpD_KpnI_R: 5´-*GGT ACC* AAC CCA GAT CAT TGG TCT ATA CTT T-3´ (*Kpn*I) |
| FTN_1072 (pSK010) | | FTN1072_F: 5´-*CAT ATG* CGT ATA TTA GTT ACA ACT T-3´ (*Nde*I) and FTN1072_R: 5´-*GAA TTC* TTA TTT ATA AGT GTT AGT TAG ATT A-3´ (*Eco*RI) |
| FTL_0879 (pJEB931) | | FTL0879_NdeI_F: 5´-*CAT ATG* CGT CTA TTA GTT ACA ACT T-3´ (*Nde*I) and FTL0879_EcoRI_R: 5´-*GAA TTC* TTA TTT ATA AGT GTT AGT CAG ATC A-3´ (*Eco*RI) |

The nucleotide sequences in italics represent the incorporated *Nde*I, *Kpn*I and *Eco*RI restriction sites used for cloning of the PCR amplified DNA fragments. Underlined sequences indicate complementary sequences in the overlap PCR primers. In primers used to generate amino acid substitutions, the nucleotides substituted are indicated in boldface. To optimize expression, these substitutions were adapted according to the codon usage preferences of *F. tularensis* (http://www.kazusa.or.jp/codon).
